# Anthocyanins Delay D‐Galactose–Induced Mouse Liver Aging by Regulating the NF‐κB/IKK Signaling Pathway

**DOI:** 10.1002/fsn3.70161

**Published:** 2025-04-17

**Authors:** Jie Wei, Zhi Tan, Guozhen Huang, Yonglian Zeng, Shilian Chen, Guandou Yuan, Songqing He, Yi Zhou

**Affiliations:** ^1^ Division of Hepatobiliary Surgery The First Affiliated Hospital of Guangxi Medical University Nanning Guangxi China; ^2^ Key Laboratory of Basic and Clinical Application Research for Hepatobiliary Diseases of Guangxi Nanning Guangxi China; ^3^ Guangxi Key Laboratory of Immunology and Metabolism for Liver Diseases Nanning Guangxi China

**Keywords:** aging, anthocyanins, inflammation, liver, NF‐κB/IKK signaling pathway

## Abstract

Aging is an intricate pathophysiological phenotype. It is the result of the combined action of various inflammatory factors and cytokines. Aging is closely related to inflammation, apoptosis, tumors, and other diseases. Anthocyanins are a kind of natural flavonoid, mainly contained in plant fruits such as bilberry, grape, purple sweet potato, and so on. These flavonoids have antioxidation, antiaging, and anti‐inflammatory properties. It has been found that anthocyanins can effectively delay liver, ovary, and other organ aging. However, the biological mechanism by which anthocyanins alleviate aging phenotypes is still poorly understood. To simulate liver aging in mice, D‐galactose was injected daily at 800 mg/kg to accelerate aging, and anthocyanins at 20 or 40 mg/kg were given as intervention treatments. The antiaging effect of anthocyanins was evaluated by body weight, inflammatory markers, and aging markers. Serum ALT and AST levels were measured, and liver histology was assessed using hematoxylin–eosin staining. In addition, we explored the molecular mechanism of anthocyanins delaying liver aging by detecting the expression levels of NF‐κB/IKK signaling protein molecules. Our results indicate that anthocyanins can effectively delay mouse liver senescence induced by D‐galactose. Analyses by Western blot demonstrated that anthocyanins inhibited the NF‐κB/IKK signaling pathway, thereby inhibiting inflammation. In vitro, anthocyanins attenuate the D‐galactose (D‐gal)–induced aging in AML12 cells, as indicated by reduced aging‐associated p21 and p16. Anthocyanins can similarly inhibit the NF‐κB/IKK signal pathway in D‐gal–induced aging in AML12 cells. Based on these findings, anthocyanins reduce liver aging in mice by regulating the NF‐κB/IKK pathway.

## Graphical abstract

Anthocyanins are expected to be an effective substance for aging intervention. For the first time, we confirmed that the protective anti‐aging effects of anthocyanins in liver and AML12 cells are mediated by inhibiting the inflammatory response through the regulation of the NF‐κB/IKK signaling pathway.

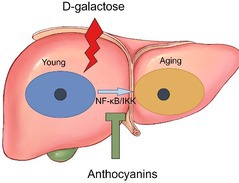

## 1 Introduction

At present, we are rapidly entering an aging society; the elderly population is increasing. By 2050, approximately 22% of the global population will be aged 60 or above (Keller et al. 2023). Aging‐related health problems have gradually become the focus of global attention. The value of exploring the molecular mechanism of cell senescence, such as the correlation between delayed senescence cell transformation and tumor occurrence, and promoting aging and tumor immune effects, deserves the attention of clinicians. Liver aging leads to the imbalance of metabolic activities at the level of molecules, tissue cells, and even organs, which leads to various abnormal pathophysiological changes. Aging‐related liver injury is generally irreversible and lacks effective treatment. Liver aging not only increases the incidence of liver diseases such as chronic hepatitis, cirrhosis, and liver tumors, but is also accompanied by a high incidence of liver failure after liver surgery (Wei et al. 2017). Aging encompasses intricate biological phenomena influenced by numerous genes and mechanisms (Yang et al. 2020). Currently, the biological processes of liver aging have not been fully elucidated, which makes reversing or delaying aging a great challenge for life science research. The D‐galactose–induced animal aging model is simpler, more cost‐effective, and quicker to construct compared to natural aging models, while maintaining similar biological characteristics (Li et al. 2020; Lin et al. 2018). Therefore, the animal model is widely used in the research of aging mechanisms. Research indicates that aging is influenced by oxidative stress, chronic inflammation, and DNA damage (Huang et al. 2022). Substantial evidence indicates that various natural flavonoids, including sulforaphane (Santín‐Márquez et al. 2019), baicalein (Tripathi et al. 2022), and anthocyanin (Wang et al. 2024), significantly contribute to decelerating the aging process.

Anthocyanins, one of the flavonoids, are widely present in food, including red raspberries, black soybeans, purple berries, grapes, apples, blueberries, strawberries, and so on (Mattioli et al. 2020; Ngamsamer et al. 2022), which is a traditional Chinese herbal native compound. Previous studies (Pratyusha and Sarada 2022; Zhang and Jing 2023) indicate that anthocyanins decelerate aging, stabilize the redox system, and inhibit inflammation. Increasing evidence indicates that anthocyanins can treat various liver diseases, including alcoholic liver injury, liver tissue damage, and fibrosis (Du et al. 2023; Xiao et al. 2021). Anthocyanins exist widely in food, are easy to obtain, and have good practical value in the treatment of chronic liver diseases. Jie W et al. confirmed through animal model studies that anthocyanins delay liver aging‐related degenerative changes (Wei et al. 2017). However, further investigation is required to elucidate the underlying anti‐aging mechanism. There remains a dearth of pertinent studies investigating the mechanism by which anthocyanins combat aging.

It has been found that the NF‐κB/IKK signaling pathway regulates various pathophysiologic processes, such as inflammation, cell proliferation, differentiation, apoptosis, and aging (Prescott et al. 2021). NF‐κB is an essential regulatory factor for endogenous inflammatory activation, cell regeneration, and oxidative stress (Priester et al. 2022). IKK is a complex of IκB kinases that regulates NF‐κB activation. Upon activation, NF‐κB moves to the nucleus to regulate gene expression (Liu et al. 2021). Fafian‐Labora JA et al. confirmed that the NF‐κB/IKK signaling pathway is extensively implicated in the modulation of both innate and adaptive immune responses and exerts a significant influence on cellular senescence (Fafián‐Labora and O'Loghlen 2021). Therefore, according to previous studies, the NF‐κB/IKK pathway may play a significant role in aging. Nonetheless, there is uncertainty about the role of the NF‐κB/IKK signaling pathway in modulating the antiaging effects of anthocyanins. The research primarily examined the potential of anthocyanins in counteracting aging by modulating NF‐κB/IKK signaling and elucidating its underlying mechanism.

## 2 Results

### 2.1 Effects of Anthocyanins on Aging Mice's Liver Function and Body Weight

**FIGURE 1:**
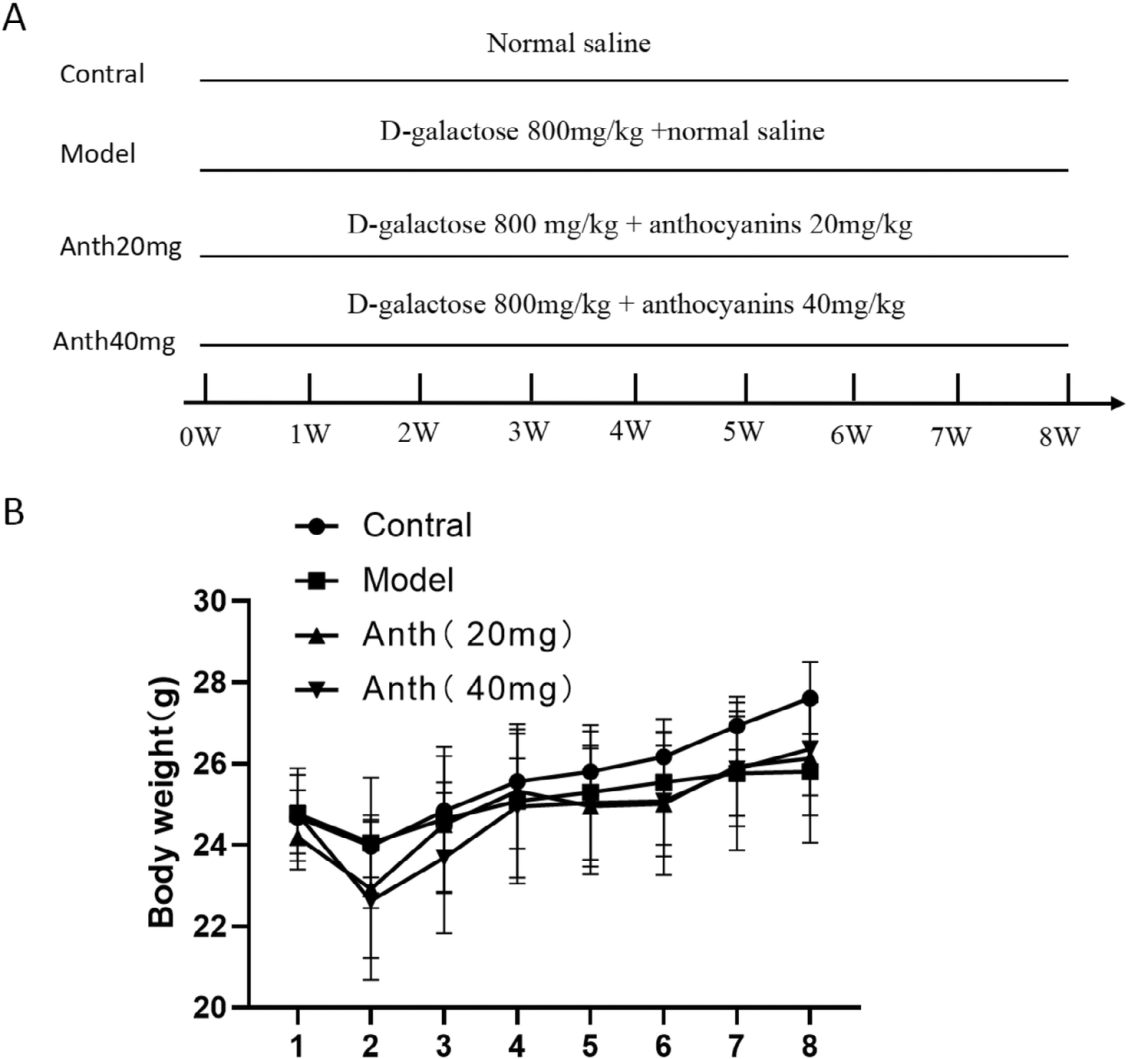
Experimental model design and body weight changes of the mouse.

**FIGURE 2:**
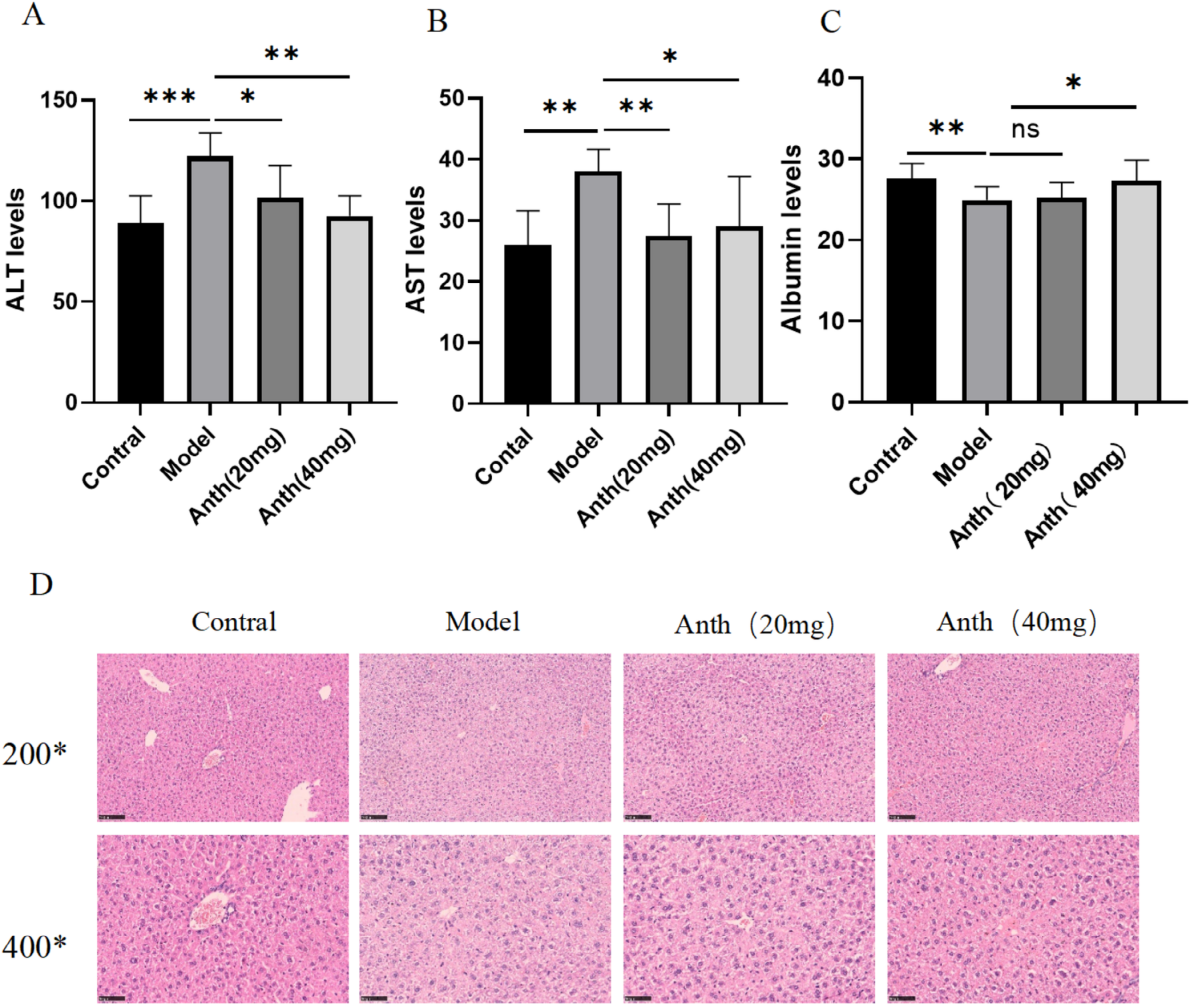
Effect of anthocyanins on D‐gal–induced liver damage.

During the 8‐week experiment, the weight fluctuations of the mice were documented on a weekly basis. The findings indicated no substantial variance in body weight among the experimental groups. At the experiment's conclusion, the control group exhibited a higher weight than the other groups (Figure 1B). ALT, AST, and albumin levels are primary indicators of liver function. Biochemical analysis indicated a significant rise in ALT and AST expression levels in the model group (*p* < 0.01 or *p* < 0.001), which were significantly lowered following treatment with both low and high doses of anthocyanins (*p* < 0.05 or *p* < 0.01) (Figure 2A,B). On the contrary, the expression level of serum albumin in the model group was decreased (*p* < 0.01), while effectively increased after treatment with high doses of anthocyanins (*p* < 0.05). There was no notable variance in the expression of albumin between the model group and the low‐dose group (Figure 2C). These results indicate that anthocyanins offer effective protection for hepatocytes and support liver function.

### 2.2 Effects of Anthocyanins on Liver Histology in Aging Mice

Liver histology examination can visually show the damage of liver tissue. Hematoxylin–eosin staining showed that more necrotic cells and inflammatory cells were infiltrated in the liver tissue of the model group. In the experimental groups, anthocyanins decreased liver cell necrosis and inflammatory cell infiltration. Anthocyanins had a good effect on alleviating liver damage in aging mice. However, no notable variance in liver damage was observed between the low‐dose and high‐dose anthocyanin groups (Figure 2D).

### 2.3 Inhibition Effects of Anthocyanins on Inflammation Levels in Aging Mice

**FIGURE 3:**
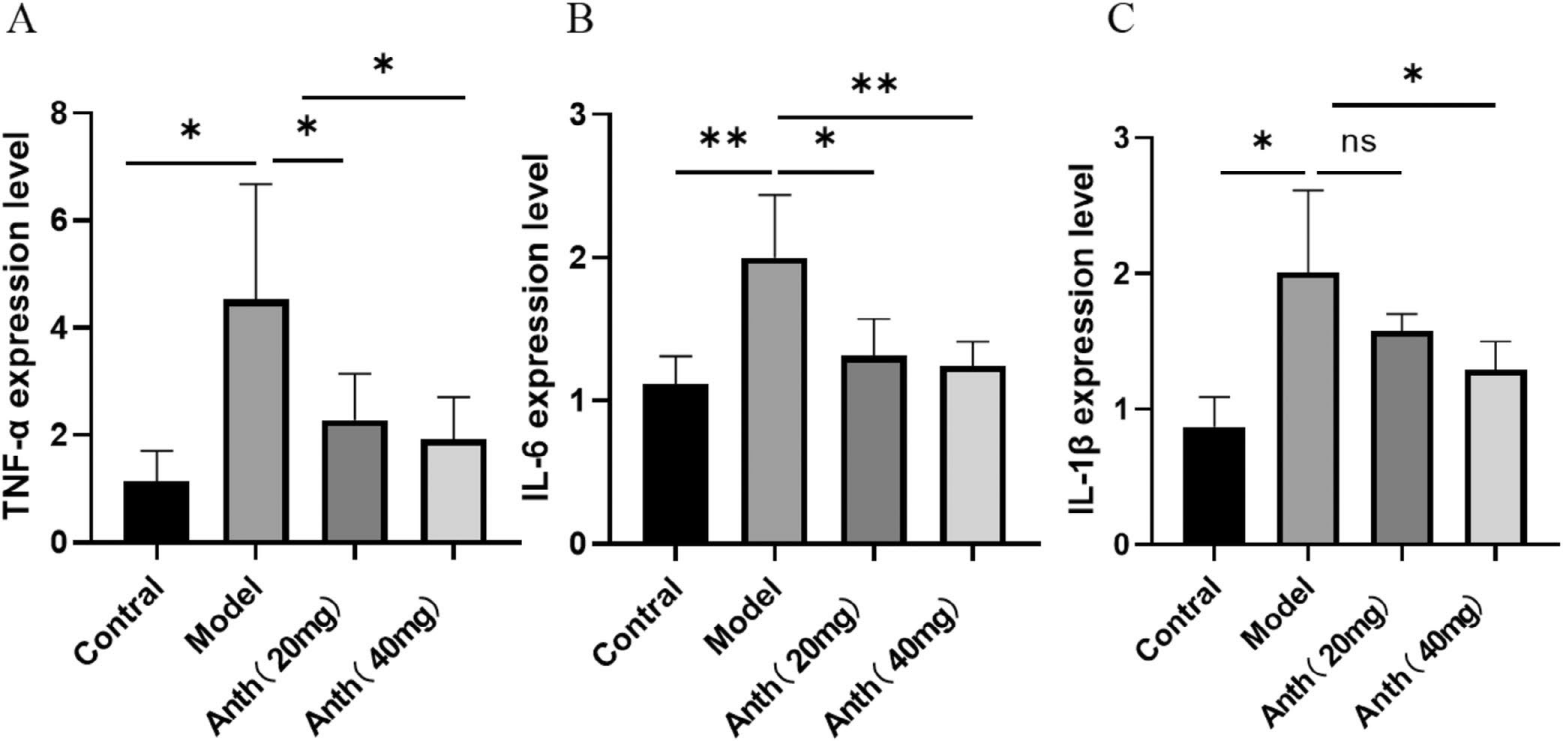
Effect of anthocyanins on inflammatory mediators in the liver tissue.

Inflammation plays a significant role in the aging process, which is a major contributor to liver function impairment. To confirm the antiaging properties of anthocyanin, quantitative real‐time PCR (qRT‐PCR) was employed to detect the key inflammatory markers IL‐1β, IL‐6, and TNF‐α in freshly collected liver tissue from mice in each experimental group. The study revealed a significant increase in IL‐1β, IL‐6, and TNF‐α expression levels in the model group (*p* < 0.05 or *p* < 0.01), which were substantially reduced after anthocyanins treatment (*p* < 0.05 or *p* < 0.01), except for IL‐1β in the Anth 20‐mg group (Figure 3A–C). The results of the experiment indicate that anthocyanins demonstrate positive effects in reducing inflammation.

### 2.4 The Effects of Anthocyanins on p16, p21, and SA‐β‐Gal of Liver in Aged Mice

**FIGURE 4:**
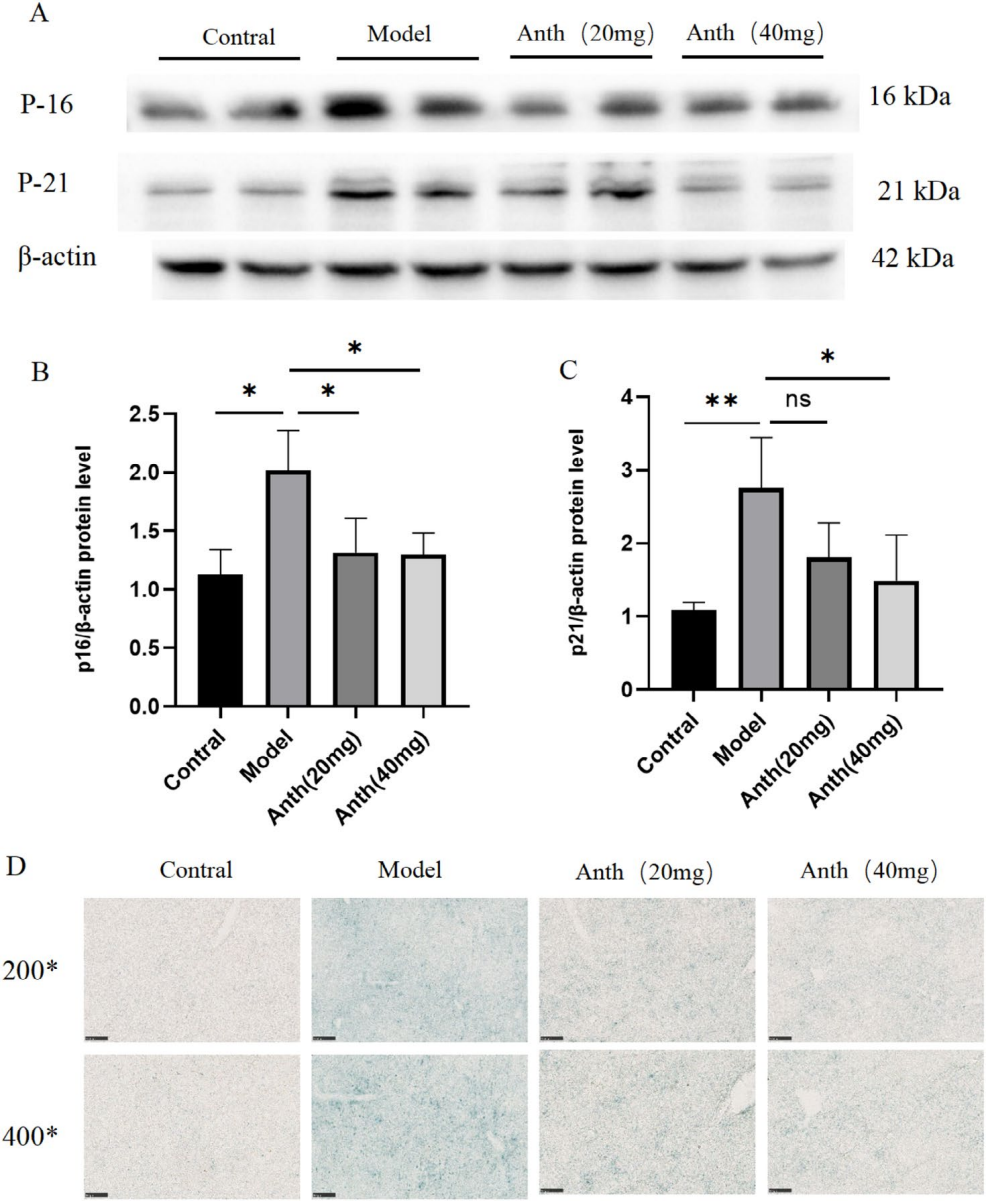
Effects of anthocyanins on D‐gal–induced cell senescence.

Protein expression levels of p16 and p21 were measured using the western blot technique. The model group exhibited significantly elevated protein expression levels of p16 and p21 compared to the control group (*p* < 0.05 or *p* < 0.01) (Figure 4A–C). Anthocyanins at doses of 20 and 40 mg/kg significantly reduced p16 expression compared to the model group (*p* < 0.05) (Figure 4A,B). The 40 mg/kg dose of anthocyanins significantly decreased p21 expression compared to the model group (*p* < 0.05). There was no significant difference in p21 expression levels between the Anth 20 mg and model groups (Figure 4A,C). SA‐β‐Gal staining revealed a significant increase in SA‐β‐Gal expression in the model group, which was notably reduced after anthocyanins treatment (Figure 4D). The above results showed that anthocyanins delayed the process of liver aging in mice.

### 2.5 Modulation of DNA Damage in the Aging Mouse Liver by Anthocyanins

**FIGURE 5:**
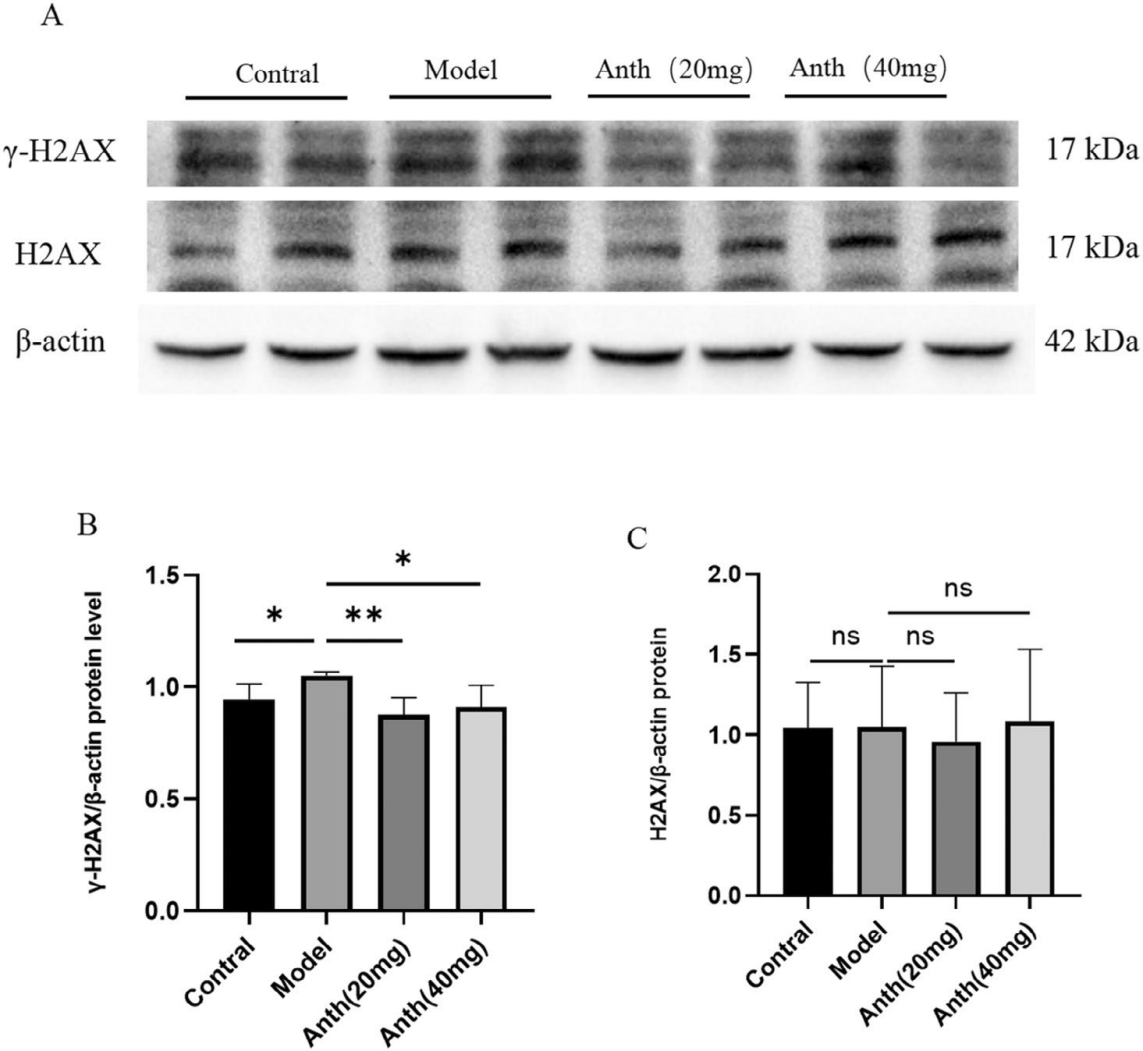
Effect of anthocyanins on D‐gal–induced DNA damage in mouse liver tissues.

We performed western blot analysis to evaluate the expression levels of γ‐H2AX and H2AX. The levels of γ‐H2AX and H2AX expression are depicted in Figure 5. The model group exhibited a significant increase in γ‐H2AX expression compared to the control group (*p* < 0.05) (Figure 5A,B). Anthocyanins at 20 and 40 mg/kg significantly reduced γ‐H2AX expression levels compared to the model group (*p* < 0.05 or *p* < 0.01) (Figure 5A,B). However, no notable variance was observed in the levels of H2AX expression among the various experimental groups (Figure 5A,C). The experimental results indicate that anthocyanins exhibit a favorable protective impact on DNA damage.

### 2.6 Anthocyanins Delay Liver Aging in Mice by Regulating NF‐κB/IKK Signaling Pathway

**FIGURE 6:**
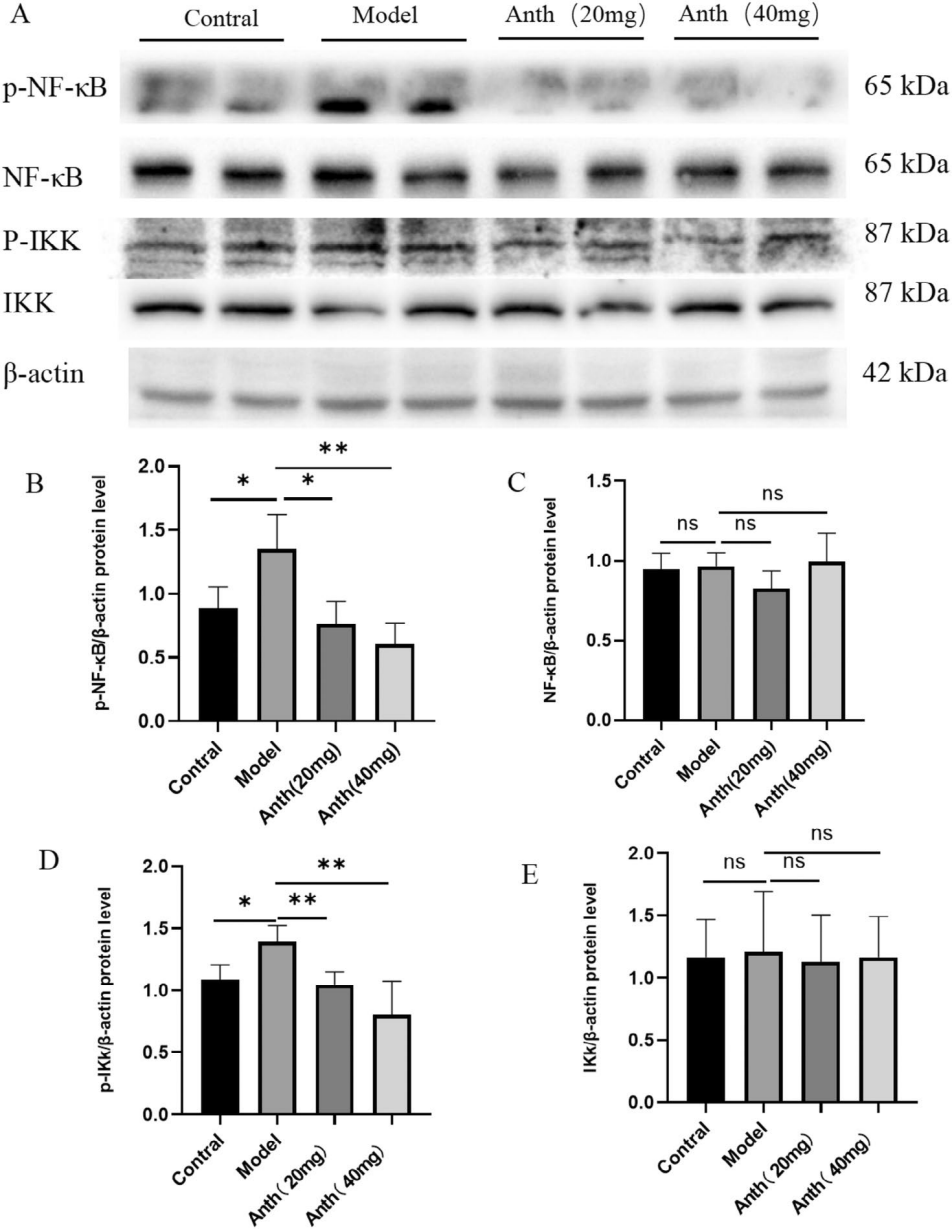
Effect of anthocyanins on the protein expression of NF‐κB/IKK signaling pathway.

We examined the influence of anthocyanins on liver aging in mice via the NF‐κB/IKK signaling pathway by performing western blot analysis to quantify the levels of NF‐κB, p‐NF‐κB, IKK, and p‐IKK proteins. Figure 6 illustrates the expression levels of p‐NF‐κB, NF‐κB, p‐IKK, and IKK. The study revealed a significant rise in p‐NF‐κB and p‐IKK expression in the model group, which was markedly reduced in the anthocyanin‐treated groups (*p* < 0.05 or *p* < 0.01) (Figure 6A,B,D). No significant differences in p‐NF‐κB and p‐IKK expression levels were observed between the anthocyanin‐treated groups and the control group (Figure 6A,B,D). The expression levels of NF‐κB and IKK did not show any notable variances across the different groups (Figure 6A,C,E). In other words, anthocyanins have been found to suppress the activation of important components in the NF‐κB/IKK signaling pathway and demonstrate a protective effect against age‐related liver damage in mice.

### 2.7 The Effects of Anthocyanins on p16 and p21 in AML12 Cells

**FIGURE 7:**
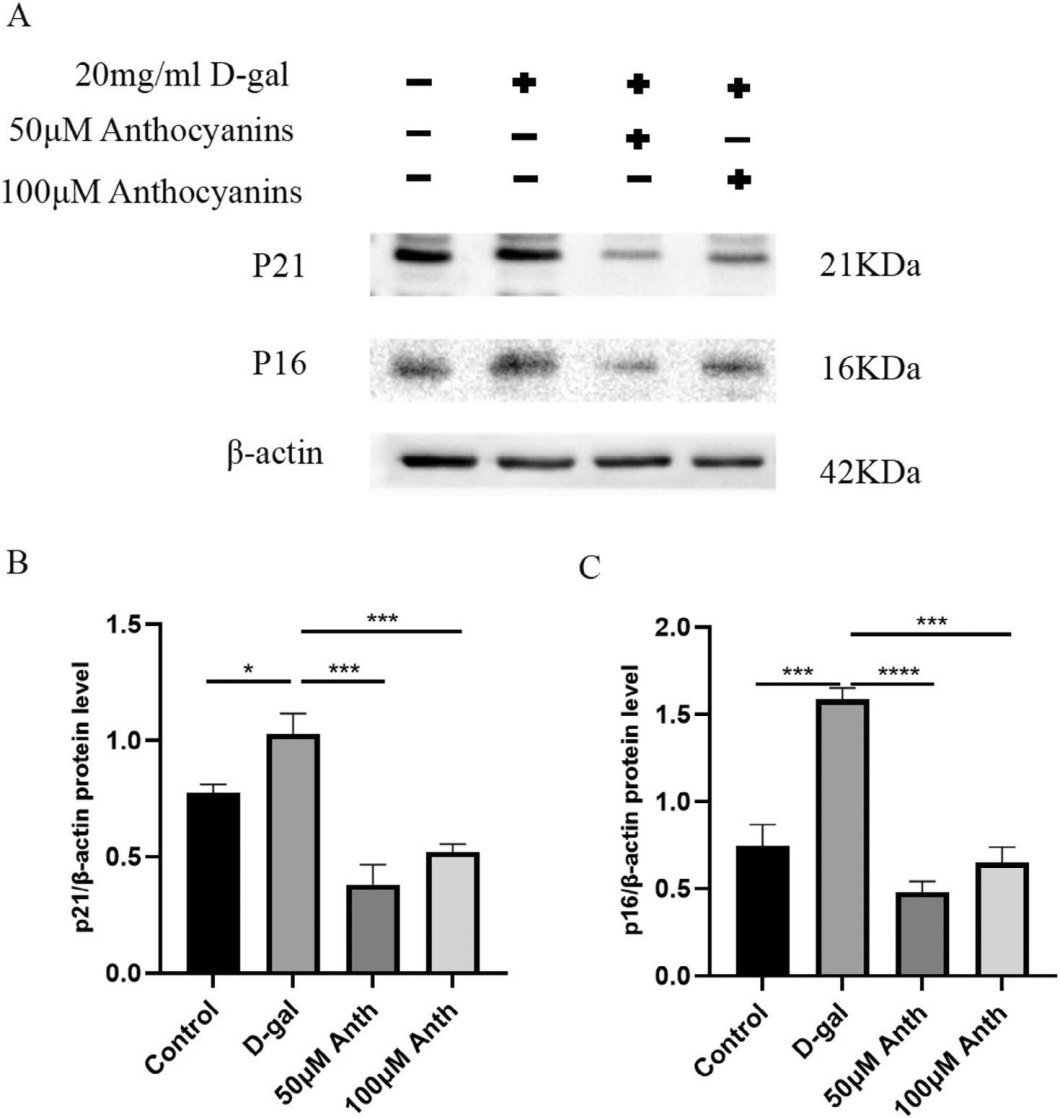
The effects of anthocyanins on p16 and p21 in AML12 cells.

To identify the effect of anthocyanins on cellular aging in cultured AML12 cells, we evaluated aging markers, including p16 and p21 protein expression. Compared with the control group, the protein expression levels of p16 and p21 were significantly higher in the D‐gal group (*p* < 0.01 or *p* < 0.005) (Figure 7A–C). Fifty and 100 μM of anthocyanins significantly reduced the expression of p21 (*p* < 0.005) (Figure 7A,B). Compared with the D‐gal group, 50 and 100 μM of anthocyanins similarly reduced the expression of p16 (*p* < 0.005 or *p* < 0.001) (Figure 7A,C). The above results showed that anthocyanins attenuate D‐gal‐induced aging in AML12 cells.

### 2.8 Anthocyanins Attenuate D‐Gal–Induced Aging in AML12 Cells by Regulating NF‐κB/IKK Signaling Pathway

**FIGURE 8:**
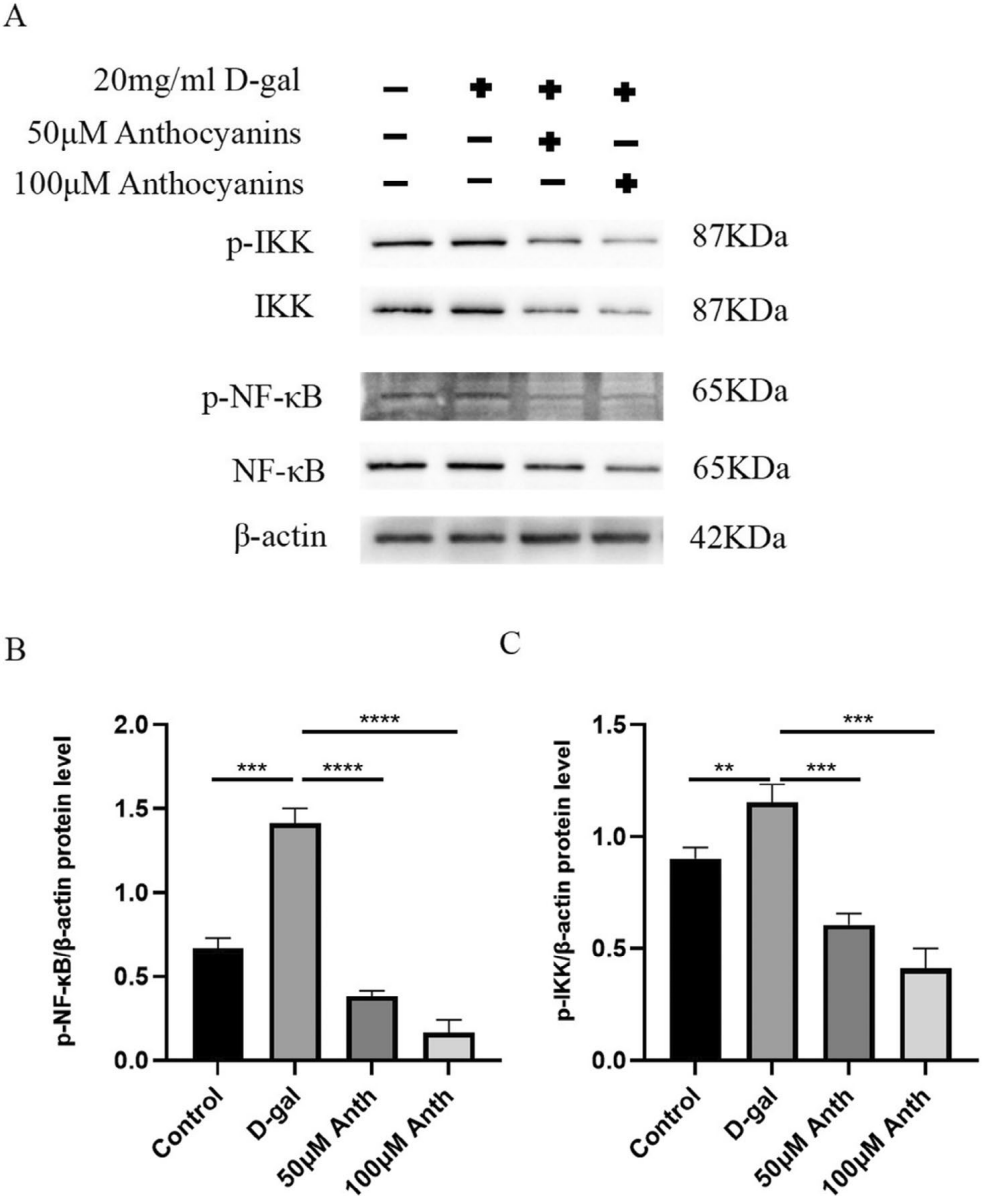
Anthocyanins attenuate D‐gal–induced aging in AML12 cells by regulating NF‐κB/IKK signaling pathway.

In vivo experiments, we have demonstrated that anthocyanins attenuate aging by inhibiting the NF‐κB/IKK pathway. In vitro, we also examined the expression of associated proteins in the NF‐κB/IKK pathway. Figure 8 shows the expression levels of p‐NF‐κB, NF‐κB, p‐IKK, and IKK (Figure 8A). It was found that the expression of p‐NF‐κB and p‐IKK was significantly elevated in the D‐gal group, while the expression was significantly reduced in the anthocyanins‐treated group (*p* < 0.01 or *p* < 0.005 or *p* < 0.001) (Figure 8A–C).

## 3 Discussion

Aging is a pathophysiological phenotype characterized by genetic material damage, cell cycle and nutrient metabolism disorders, accompanied by the production of cytoplasmic chromatin fragments (CCFs), secretion of a variety of inflammatory factors and cytokines, and cell cycle arrest (Huang et al. 2022). On the one hand, physiological aging is an important basis for tissue and organ damage repair and embryonic development (Hernandez‐Segura et al. 2017). On the other hand, pathological aging is the root of organ atrophy, functional decline, regenerative disorders, tumors, and many other diseases (Gonzalez‐Meljem and Martinez‐Barbera 2024; Xu et al. 2021). Aging occurs due to a range of factors that cause damage. It is now generally accepted that oxidative stress damage and inflammation are important factors in promoting aging. The underlying biological mechanisms of aging are complex and unexplained, which pose great challenges to the intervention of aging. However, research findings have suggested that various traditional indigenous substances exhibit distinct therapeutic properties in retarding the aging process (Shen et al. 2017). Anthocyanins are an important bioactive natural substance, which can delay aging through a variety of signaling pathways. For example, Lee GH et al. found in their studies that anthocyanins can attenuate endothelial dysfunction in aging rats by regulating uncoupling of nitric oxide synthase (Lee et al. 2020). A diet abundant in anthocyanins can impede the aging of vascular endothelial cells and prevent the occurrence of cardiovascular diseases (Dong et al. 2022). Wang B et al. reported that anthocyanins could exert an anti‐aging therapeutic effect through antioxidation, anti‐inflammation, inhibition of insulin/IGF‐1 signaling (IIS) and other mechanisms (Wang et al. 2024). Chen S et al. confirmed that long‐term intake of anthocyanin alleviated liver injury and abnormal amino acid metabolism in D‐galactose–induced aging mice (Chen et al. 2022). In our study, we also demonstrated the biopotency of anthocyanins in delaying D‐galactose–induced liver aging in mice. The anthocyanin‐treated group exhibited significantly lower expression levels of SA‐β‐Gal, p16, and p21 compared to the model group. In vitro, we obtained the same results with elevated p16 and P21 expression in D‐gal–treated AML12 cells. The experimental group exhibited a higher presence of necrotic cells and inflammatory cell infiltration. However, subsequent administration of anthocyanins resulted in a significant reduction in ALT, AST, and inflammatory marker levels. This indicates that anthocyanin alleviated liver cell damage. Previous research has demonstrated that anthocyanins are effective in treating fatty liver disease, liver fibrosis, and other chronic liver conditions (Zhang et al. 2021a; Zhu et al. 2022). However, it is undeniable that the protective mechanism of anthocyanins in the aging process has not been elucidated so far.

The aging phenotype is associated with a persistent and widespread inflammatory response. Numerous evidences have confirmed that inflammation is an important factor in inducing aging (Arosio et al. 2023; Li et al. 2023), and excessive inflammation will lead to the imbalance of homeostasis. There is an interaction between inflammation and aging. Senescent cells secrete a variety of cytokines, that is, senescence‐related secretion phenotypes, which activate multiple inflammatory cells to induce a chronic inflammatory response (Schmitt et al. 2023). In contrast, long‐term systemic chronic inflammation releases a large number of inflammatory factors, weakens immune function, and the ability to clear senescent cells, which are important factors in promoting aging (Li et al. 2023). Besides, genomic damage and instability are important factors that promote cell senescence (Zhao et al. 2023). Extensive inflammatory response leads to the accumulation of DNA damage, which induces aging and a variety of age‐related diseases (Pezone et al. 2023). Studies show that intervention of chronic inflammation will provide a new target for antiaging therapy (Neves and Sousa‐Victor 2020), and blocking TNF‐α signaling pathway could effectively improve the cognitive function of aging rats (Ma et al. 2015). Arosio B et al. identified that IL‐1β and IFN‐γ, common inflammatory markers, significantly contribute to aging (Arosio et al. 2023). In addition, there were scholars who also reported that the application of platelet factors could reduce the level of nervous system inflammation in aging mice and improve their cognitive ability (Schroer et al. 2023). This study found that anthocyanin treatment significantly reduced liver tissue inflammation and aging marker expression compared to the model group. These reflect that anthocyanins may exert antiaging effects by inhibiting inflammation levels. The NF‐κB/IKK pathway is a key proinflammatory signaling mechanism extensively involved in gene transcription regulation and closely associated with inflammation control (Priester et al. 2022). IKK, an IκB kinase complex, significantly contributes to NF‐κB activation (Priester et al. 2022). After phosphorylation, NF‐κB is transferred to the nucleus to regulate gene transcription, promote the synthesis of various cytokines, and promote inflammation (Liu et al. 2021). Inhibiting NF‐κB pathway activation significantly decreases inflammation levels. The inhibition of the NF‐κB/IKK signaling pathway by Wnt4 significantly reduces the expression levels of key inflammatory factors, including IL‐6, TNF‐α, and IL‐1β, thereby alleviating LPS‐induced inflammation in pulp cells (Ni et al. 2023). Inhibiting the NF‐κB/IKK signaling pathway activation suppresses inflammation from various causes and alleviates inflammatory disease symptoms, offering a novel therapeutic approach (Bains et al. 2022; Gan et al. 2023; Xie et al. 2024). Therefore, we infer that the NF‐κB/IKK signaling pathway may influence the aging process by regulating the inflammatory response. Previous research supports the view that the NF‐κB/IKK signaling pathway plays a crucial role in aging (Kolesnichenko et al. 2021). Zhang L et al. demonstrated that inhibiting the NF‐κB/IKK signaling pathway using the novel small molecule SR12343 significantly decreased SA‐β‐gal expression in aging mice, thereby mitigating aging (Zhang et al. 2021b). Fafián‐Labora JA et al. demonstrated that the aging process relies on the activation and regulation of the classical NF‐κB/IKK signaling pathway, and that inhibiting NF‐κB or IKK halts aging (Fafián‐Labora and O'Loghlen 2021).

To elucidate the molecular mechanisms and downstream pathways involved in senescence and examine the association between anthocyanins, the NF‐κB/IKK signaling pathway, and aging. We used western blot to measure the protein expression levels of the NF‐κB/IKK signaling pathway under various experimental conditions. Simultaneous anthocyanin administration effectively reduces p‐NF‐κB and p‐IKK expression levels. The expression levels of p‐NF‐κB and p‐IKK align with the patterns observed in aging markers (SA‐β‐gal, p16, p21, and γ‐H2AX), inflammation, and liver damage. These results showed that mice treated with anthocyanins had less aging and a better liver function index than those in the model group. It is evident that the NF‐κB/IKK signaling pathway may represent a crucial target for anthocyanins in ameliorating the aging process. Several studies concur that anthocyanins mitigate inflammation by inhibiting NF‐κB signaling pathway activation and alleviating symptoms of various inflammatory conditions. Chen S et al. found that anthocyanins mitigate oxidative stress and inflammation in the brain tissue of D‐galactose‐induced aging rats by inhibiting the NF‐κB signaling pathway (Chen et al. 2019). Ye Y et al. reported that anthocyanins reduce osteoarthritis by acting on the NF‐κB signaling pathway (Ye and Zhou 2023). Chen J et al. discovered that anthocyanins can inhibit the IKK/NF‐κB pathway, thereby reducing the activation of downstream inflammatory factors and enhancing hepatocyte structure and function in high‐fat diet‐/streptozotocin‐induced T2DM mice (Chen and Meng 2022). We hypothesize that anthocyanins may mitigate liver tissue inflammation in aging mice by inhibiting the NF‐κB/IKK signaling pathway, thereby protecting liver function and delaying aging. It will become a potential direction to study liver aging, and even a new approach to treat aging.

Liver aging presents significant challenges for preventing and treating liver diseases, such as inhibiting liver regeneration and decreasing surgical tolerance. In this study, we found the potential role of anthocyanins in alleviating liver aging. Exploring the application of anthocyanins in antiaging is highly significant. While the exact mechanism by which anthocyanins delay liver aging remains unclear, this study offers a novel perspective for future aging intervention and research. At the same time, our research also has the following several limitations. Firstly, the process of aging is systemic, and we focus on the aging of the liver without studying other organs. Secondly, in the current study, we did not intervene in the NF‐κB/IKK signaling pathway, which is our next research plan. Thirdly, the dose of anthocyanins was mainly selected based on the results of previous studies in this study, and whether a higher dose will achieve better therapeutic effects still needs further research. In addition, during the experiment, the experimental mice showed relatively little weight gain compared to the naturally grown mice. In addition to the influence of D‐galactose, gavage‐induced esophageal damage affecting the feeding of mice is also an important factor to be considered. In future studies, we need to fully consider the harmful effect of gavage on the feeding and nutritional status of mice, which may lead to the bias of the study results.

Our findings indicate that the protective antiaging effects of anthocyanins are mediated by inhibiting the inflammatory response through the regulation of the NF‐κB/IKK signaling pathway. Anthocyanins are expected to be an effective substance for aging intervention. However, the specific mechanism of action and effective dose need to be further explored.

## 4 Materials and Methods

### 4.1 Materials

Anthocyanins purchased from Yuanye Bio (CAS#4852‐22‐6), purity > 95%. D‐galactose is purchased from Sigma‐Aldrich (Lot#BCCC5024).

### 4.2 Cell Cultures

Mouse AML12 cells were obtained from the Stem Cell Bank, Chinese Academy of Sciences, China. AML12 cells were cultured in Dulbecco's modified Eagle medium (DMEM)/F12 (Gibco, 11,330) supplemented with 40 ng/mL dexamethasone (Sigma, D4902‐100 mg), 10% fetal bovine serum (FBS), and 1% insulin–transferrin–selenium (ITS) liquid media supplement (Sigma, I3146). AML12 cells were treated with D‐gal for 48 h. Two doses of anthocyanins were added 24 h after the onset of the D‐gal challenge.

### 4.3 Animals

Thirty‐two male C57BL/6J mice (6–8 weeks old, 20–24 g) were sourced from the Animal Center of Guangxi Medical University (Nanning, China). The experimental mice were raised in an animal house free of specific pathogens (SPF) in the Medical Laboratory Animal Center of Guangxi Medical University. The 12‐h light/dark cycle was carried out. The animal house maintained a constant temperature of 23°C ± 1°C and humidity of 55% ± 5%, with mice having unrestricted access to food, water, and activities. Thirty‐two mice were randomly divided into four groups (*n* = 8 per group): (i) Control group (normal saline treatment); (ii) Model group (D‐galactose 800 mg/kg in saline); (iii) Anth 20 mg group (D‐galactose 800 mg/kg + anthocyanins 20 mg/kg in saline); (iv) Anth 40‐mg group (D‐galactose 800 mg/kg + anthocyanins 40 mg/kg in saline) (Figure 1A). D‐galactose was administered by subcutaneous injection, and anthocyanins were administered by gavage. All medications were used for 8 weeks, and body weight was recorded weekly. After 8 weeks, the mice were euthanized, whole blood and liver were obtained, and the liver index was calculated.

### 4.4 Measurements of ALT/AST (Plasma)

ALT, AST, and serum albumin expression levels were detected by a biochemical analyzer.

### 4.5 Histopathological Examination and Evaluation of Liver

All liver tissue was obtained from the same site. Liver tissue was fixed in 10% formaldehyde for 24–48 h, dehydrated using a gradient alcohol automatic dehydrator (Leica, ASP300S), embedded in paraffin, sectioned into 4‐μm slices, and stained with hematoxylin–eosin for microscopic examination. The thick paraffin slices (4 μm) were dewaxed with xylene three times (5 min each), fully hydrated with an alcohol gradient (5 min each), and then dewaxed with water. Paraffin sections were stained with hematoxylin at room temperature for 1–2 min, rinsed with tap water for 5 min, and then redyed with a 0.5% eosin solution for 20–30 s. Stained paraffin sections were dehydrated in an alcohol gradient and sealed with xylene transparent and neutral resin. Liver injury was observed by optical microscope (Olympus Corporation, CX‐23) (magnification, ×200 and ×400).

### 4.6 SA‐β‐Gal Staining

Fresh liver tissue was embedded in OCT, quickly fixed with liquid nitrogen, and tissue slices (8 μm) were cut using a frozen microtome (Leica, CM1950). The frozen tissue slices were rewarmed and then soaked in PBS three times, each time for no less than 5 min. Draw a suitable circle around the liver tissue, add an appropriate amount of β‐galactosidase stain fixing solution, and fix at room temperature for no less than 15 min. Soak the washing tissue with PBS for three times for not less than 5 min each time. PBS was absorbed, add an appropriate amount of dyeing solution, and incubate at 37°C overnight. Automatic scanning machine (Hamamatsu, NanoZoomer S60) after sealing with sealing liquid.

### 4.7 Quantitative RT‐PCR

Quantitative real‐time PCR analysis quantifies inflammatory factor expression levels. Primers were synthesized and designed for mouse cytokine analysis: interleukin‐1(IL‐1β), left 5'‐CCGTGGACCTTCCAGGATGA‐3' and right 5'‐GGGAACGTCACACACCAGCA‐3'; interleukin‐6 (IL‐6), left 5'‐CCTCTGGTCTTCTGGAGTACC‐3' and right 5'‐ACTCCTTCTGTGACTCCAGC‐3'; The tumor factor‐α (TNF‐α), left 5'‐GTAACCCGTTGAACCCCATT‐3' and right 5'‐CCATCCAATCGGTAGTAGCG‐3'. 18S served as the internal control.

### 4.8 Western Blot Analysis

The expression levels of P16, P21, γ‐H2AX, H2AX, NF‐κB, p‐NF‐κB, IKK, and p‐IKK proteins were analyzed using western blot. The primary antibodies used included NF‐κB, p‐NF‐κB, IKK, p‐IKK, P16, P21, γ‐H2AX, and H2AX. Horseradish‐conjugated goat anti‐rabbit secondary antibody (1:10,000; cat. no. SA00001‐2; ProteinTech Group Inc.).

### 4.9 Statistical Processing

Data were presented as mean ± standard deviation (SD) and analyzed with GraphPad Prism 8.0 software. One‐way ANOVA with Tukey's posttest was conducted using GraphPad Prism 8 for multiple comparisons. Significance was determined by *p*‐values less than 0.05.

## Author Contributions

**Jie Wei:** formal analysis (equal), investigation (lead), project administration (equal), supervision (equal), writing – original draft (lead). **Zhi Tan:** investigation (equal), project administration (lead). **Guozhen Huang:** investigation (supporting), project administration (supporting), validation (lead). **Yonglian Zeng:** data curation (lead), formal analysis (lead), validation (equal). **Shilian Chen:** data curation (equal), software (lead), validation (equal). **Guandou Yuan:** investigation (supporting), project administration (supporting), visualization (lead), writing – review and editing (equal). **Songqing He:** conceptualization (equal), funding acquisition (lead), resources (lead), supervision (equal). **Yi Zhou:** conceptualization (lead), funding acquisition (equal), project administration (equal), resources (equal), supervision (lead), writing – review and editing (equal).

## Ethics Statement

All animal procedures performed in this study have been approved by the Medical Ethics Committee of the First Affiliated Hospital of Guangxi Medical University (Approval No. 2024‐E143‐01).

## Conflicts of Interest

The authors declare no conflicts of interest.

## Data Availability

Data are available on request from the authors.
